# Frontal increase of beta modulation during the practice of a motor task is enhanced by visuomotor learning

**DOI:** 10.1038/s41598-021-97004-0

**Published:** 2021-08-31

**Authors:** E. Tatti, F. Ferraioli, J. Peter, T. Alalade, A. B. Nelson, S. Ricci, A. Quartarone, M. F. Ghilardi

**Affiliations:** 1 CUNY School of Medicine, 160 Convent Avenue, Harris Hall Room 008, New York, NY 10031 USA; 2 DIBRIS University of Genova, 16145 Genoa, Italy; 3 Department of Biomedical, Dental Sciences and Morphological and Functional Images, University of Messina, 98125 Messina, Italy

**Keywords:** Cognitive control, Motor cortex, Long-term potentiation, Short-term potentiation, Cortex, Electroencephalography - EEG, Human behaviour

## Abstract

Movement is accompanied by beta power changes over frontal and sensorimotor regions: a decrease during movement (event-related desynchronization, ERD), followed by an increase (event-related synchronization, ERS) after the movement end. We previously found that enhancements of beta modulation (from ERD to ERS) during a reaching test (*mov*) occur over frontal and left sensorimotor regions after practice in a visuo-motor adaptation task (ROT) but not after visual learning practice. Thus, these enhancements may reflect local cumulative effects of motor learning. Here we verified whether they are triggered by the learning component inherent in ROT or simply by motor practice in a reaching task without such learning (MOT). We found that beta modulation during *mov* increased over frontal and left areas after three-hour practice of either ROT or MOT. However, the frontal increase was greater after ROT, while the increase over the left area was similar after the two tasks. These findings confirm that motor practice leaves local traces in beta power during a subsequent motor test. As they occur after motor tasks with and without learning, these traces likely express the cost of processes necessary for both usage and engagement of long-term potentiation mechanisms necessary for the learning required by ROT.

## Introduction

Movement is associated with modulation of beta oscillatory activity (13–25 Hz) over sensorimotor regions: movement preparation and execution are characterized by decreased beta power (event-related desynchronization, ERD), followed by a hefty rebound (event-related synchronization, ERS) once the movement is completed^1,2^.

Over the last decades, several frameworks have been proposed to explain the functional role of sensorimotor beta power and its movement-related modulation^3–5^. At the present time, there is a general agreement in considering beta modulation as resulting from the interplay between motor and sensory regions. Accordingly, beta ERD should reflect the activation of the motor network and the increase in corticospinal excitability^6^, while the subsequent rebound (ERS) would represent the activation of an extended network, which includes somatosensory and prefrontal regions, with the purpose of assessing and eventually updating the activated motor representations. This updating process makes it likely that movement-related beta modulation, and ERS in particular, may be linked to the engagement of long-term potentiation-(LTP) mediated mechanisms. This hypothesis is in line with a series of recent reports; first, TMS studies have shown that facilitation of corticospinal excitability elicited with iTBS also results in increased beta ERS^7^. Second, EEG studies demonstrated a progressive enhancement of sensorimotor and frontal beta ERS amplitude with motor practice^8–11^, learning^12^ and sensorimotor adaptation^13^. Third, the practice-related increases of beta modulation depth (measured from peak ERD to peak ERS) correlates with skill retention tested the following day^9^ and led to local enhancements of beta power in the post-training resting state EEG^8,14^, enhancements that vanish after both a period of quiet rest and sleep^14^. Finally, in a recent study^15^ we found that this carry-over effect was also present in movement-related beta modulation during an ensuing simple reaching test (*mov*). Indeed, after three one-hour motor learning in a task requiring implicit adaptation to a visually-rotated display (ROT), the increase of beta modulation during *mov* over frontal and contralateral sensorimotor regions was greater than after a visual learning task without motor component. This suggests that previous intensive motor learning locally enhances practice-related beta modulation increase.

Are the carry-over effects on beta modulation strictly related to motor learning or do they occur to the same degree after practice in a motor task without a learning component? To answer this question, here we investigated whether extensive motor practice in two reaching tasks, one requiring continuous visuo-motor adaptation (ROT) and the other only plain movements (MOT), would leave the same local traces on movement-related beta modulation during a successive reaching movement test (*mov*).

The two tasks are similar as they involve the sensorimotor network, but they differ in terms of activation of areas involved in visuo-motor transformation^16,17^. In particular, the early phases of adaptation in ROT are associated with increased activity of pre-supplementary motor area compared to MOT^17^ and the later phases with activation of right parietal areas^18^.

## Results

**Figure 1:**
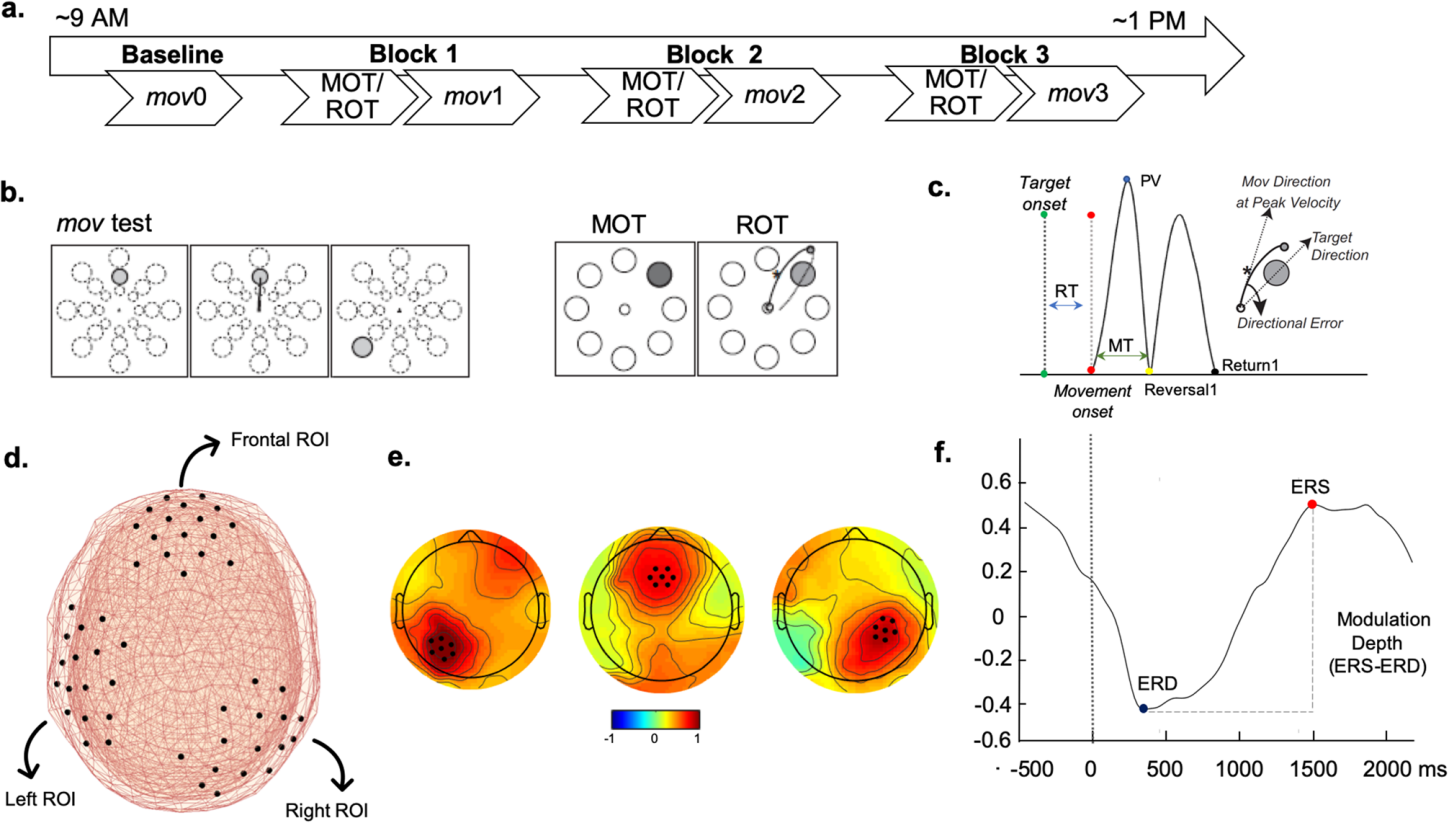
(**a**) Experimental design. Four blocks of a simple reaching test (*mov*) were interspaced by three 45-min blocks of practice of either ROT, an implicit visuo-motor learning task, or MOT, a motor reaching task with the same kinematic characteristics of ROT but without adaptation learning. (**b**) *mov* test: one of 24 targets (three distances, eight directions) appeared in unpredictable order every 3 s, ROT and MOT tasks: a circular array of 8 targets (4 cm distance) was presented on the display and one target blackened every 1.5 s in a random, unpredictable order. For both *mov* and ROT/MOT task, participants were instructed to reach the target as soon as possible, without correcting the movement and avoiding anticipation. In ROT the direction of the cursor was rotated relative to the trajectory of the movement in steps of 10°, 20° or 30°, either clockwise or counterclockwise. (**c**). Temporal profile of velocity showing the kinematic features considered for this study: RT, Reaction Time is the time difference between target appearance and movement onset; MT, Movement Time is the time difference between movement onset and return; PV, Peak Velocity is the first relative velocity maximum from onset to reversal; Directional error is the angular difference between the direction linking the center, the PV point and the target direction. (**d**,**e**) Methodology for computing beta modulation depth. (**d**) For each subject, beta ERD and ERS peak amplitude and timing were first computed on *mov*0 ROT/MOT1 over three broad regions corresponding to electrodes located on the frontal, left, and right scalp regions (**e**) The peak electrode and the six neighbor ones for both Left, Frontal and Right scalp regions defined the personalized ROIs. (**f**) Beta modulation depth is defined by the amplitude difference between the peak ERS and peak ERD in the ROI.

Two groups of healthy young subjects completed three morning sessions of intensive practice in one of two reaching tasks: ROT, where subjects constantly and implicitly adapted their movements to visual rotations of different degree or MOT, a control task kinematically equivalent to ROT but without adaptation learning. To determine the effects of task practice on general motor performance, at baseline and after the practice in the two tasks, both groups also completed a short block of *mov*, a simple reaching test with targets presented at three distances (Fig. 1).

### ROT and MOT practice differentially affect regional beta modulation amplitude

The detailed performance in the ROT and MOT tasks has been presented in previous work^14^. Briefly, in the ROT session, participants adapted to each imposed rotation by decreasing the directional error across the movements of two consecutive sets (see Supplementary Figure S1). Importantly, adaptation rates did not differ in ROT1 (71.6% ± 4.1%) and ROT3 (72.7% ± 3.4%) suggesting that learning soundly occurred and was similar in the two ROT blocks. Conversely, no major changes of directional errors were evident in MOT (Supplementary Figure S1) confirming that MOT and ROT differed in terms of visuo-motor learning. In both ROT and MOT sessions, participants also reported their subjective tiredness level at baseline and after each block. Compared to the baseline assessment, subjective tiredness score was significantly greater after the third block of ROT (ROT1 = 0.37 ± 1.39; ROT3 = 1.74 ± 1.70, Wilcoxon signed-rank test, Z = 3.58, *p* = 0.00035) but decreased in MOT (MOT1 = 1.15 ± 1.62; MOT3 = 0.07 ± 2.14, Z = − 2.51, *p* = 0.012). A direct comparison of the two tasks also showed that tiredness score in ROT3 was significantly higher than after MOT3 practice (Kruskall-Wallis test, Block1: H = 3.27, *p* = 0.071, Block3: 6.23, *p* = 0.013). Importantly, tiredness did not seem to influence adaptation rates, as indicated by the lack of significant correlation between the two (Block1: rho = 0.09, *p* = 0.66; Block 3: rho = 0.25, *p* = 0.22).

We then ascertained whether within-block changes of performance were similar in the first and last block of MOT and ROT. To allow for task comparisons unbiased from contamination of rotation learning, we focused on the kinematic data from the first and last sets of MOT1, MOT3, ROT1 and ROT3. Specifically, we computed the difference between movements in the last and first two sets as they had no imposed rotation also in ROT. As the kinematic data in ROT and MOT were not normally distributed (see “Methods” section), we used independent-samples Kruskal–Wallis and related-samples Wilcoxon-signed rank tests to assess between-tasks and between-blocks differences. Such comparisons revealed no significant within-block changes both between tasks and between blocks for reaction time, movement time and peak velocity (Supplementary Table S1). There was an average difference of less than 1° between mean directional of ROT3 and MOT3 (ROT3 First: 4.9° ± 0.8°, ROT3 Last: 5.8° ± 1.1°; MOT3 First: 5.0° ± 1.2°; MOT3 Last: 5.1° ± 1.0) that however reached statistical significance (Supplementary Table S1).

**Figure 2:**
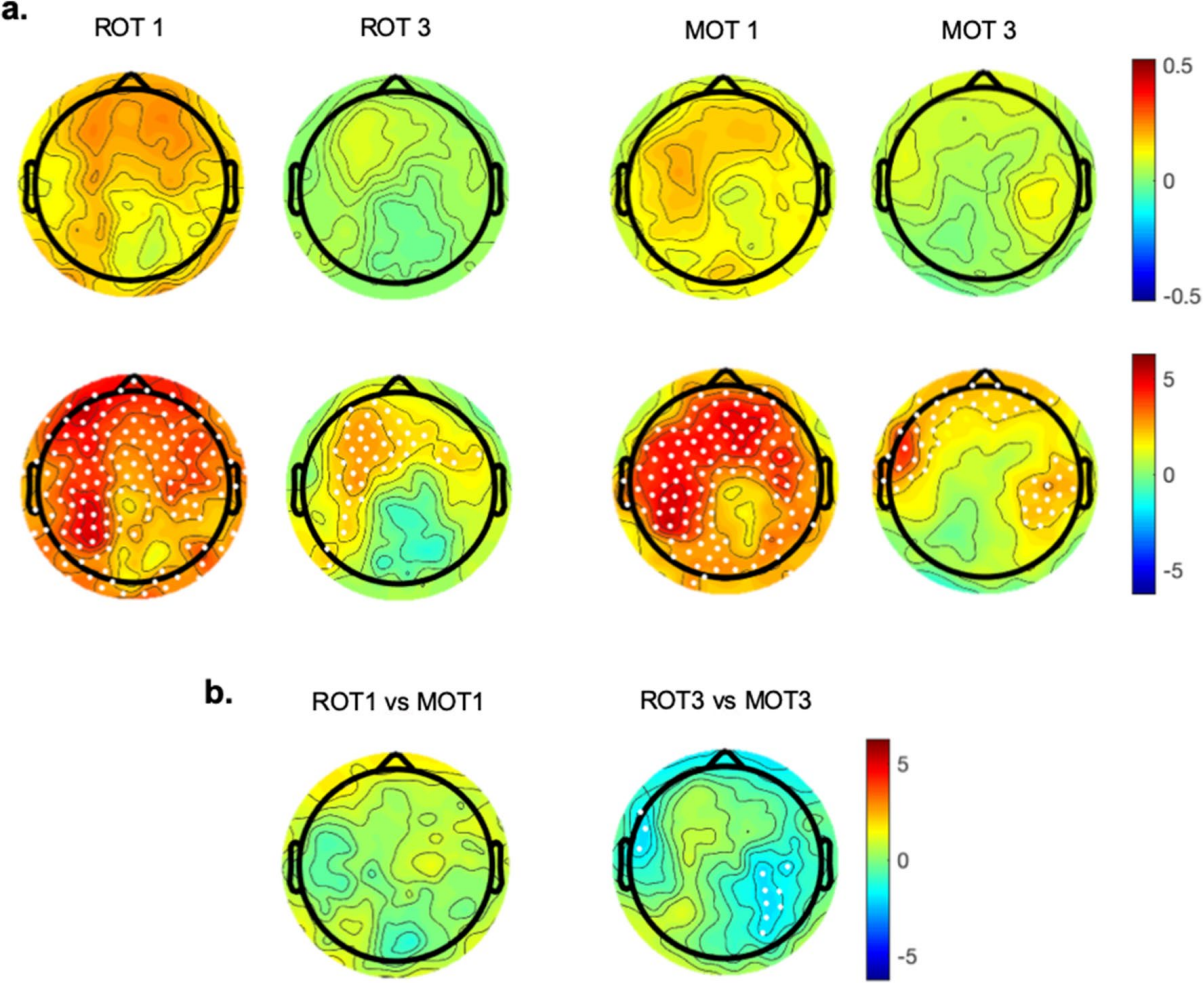
Within-block changes in average beta oscillatory activity in the ROT and MOT tasks. (**a**) Top. Topographic distribution of the within-block changes—computed as the difference between the last and first two zero-degree rotation sets—in the first and last blocks of ROT (ROT1 and ROT3) and MOT (MOT1 and MOT3). Bottom. Bonferroni-corrected permutation t-values maps. Dots indicate significant electrodes (*p* ≤ 0.01 for Block 1; *p* ≤ 0.05 for Block 3). (**b**) Maps of the Bonferroni-corrected permutation t-values comparing the within-block changes of the two tasks in the first block (ROT1 vs. MOT1) and in the last block (ROT3 vs. MOT3). Significant electrodes are reported as white dots.

We then asked whether the two types of practice left a specific hallmark in beta oscillatory activity. Therefore, we first focused on the changes of mean beta power during the tasks and compared the differences between first and last sets of movements during the first block of both MOT (MOT1) and ROT (ROT1) with Bonferroni-corrected paired t-test permutation analyses (alpha = 0.01, see “Methods” section). For both tasks, this analysis showed a considerable increase in the average beta power, with the greatest increment located over the frontal and left sensorimotor regions (significant electrodes, mean ± SD: ROT1: 17.92% ± 21.67%, t = 3.44 ± 0.67, *p* = 0.002 ± 0.002; MOT1: 14.91% ± 12.31%, t = 3.67 ± 0.76, *p* = 0.003; Fig. 2a). During the third block (MOT3 and ROT3), the magnitude of the within-block increase was reduced in both tasks, as reflected by the lack of statistical significance. Nevertheless, the same analyses with the alpha threshold lowered at 0.05 revealed a significant within-block increase in a set of electrodes located over a similar left-frontal region both in ROT3 (mean ± SD: 6.18% ± 12.44%, t = 2.19 ± 0.31, *p* = 0.024 ± 0.013) and MOT3, even though the enhancement was more localized in the prefrontal electrodes in the latter (mean ± SD: 7.77% ± 10.71%, t = 2.33 ± 0.26, *p* = 0.014). MOT3 presented also significant activity increase in electrodes over the right centro-parietal region (mean ± SD: 9.80% ± 14.19%, t = 2.22 ± 0.26, *p* = 0.025 ± 0.009, Fig. 2a). A direct comparison between the two tasks indicated that ROT and MOT displayed a similar pattern of within-block beta power increase in both blocks. However, MOT3 showed a slightly larger increase in few electrodes over the left frontal (ROT3 vs. MOT3: − 7.9%, t = − 1.96 ± 0.15, *p* = 0.026 ± 0.012) and right parietal region in (ROT3 vs. MOT3 vs.: − 9.45%, t = − 1.98 ± 0.16, *p* = 0.029 ± 0.09, Fig. 2b).

We then determined whether the within-block changes (Last-First sets) of beta modulation depth (peak ERS-ERD) equally occurred in the first and last blocks. We focused these analyses on the three ROIs, i.e., Left, Right and Frontal, defined by the electrode with the maximum value of beta modulation depth and the six neighboring ones (see “Methods” section). Due to violation of the normality assumption, we used a two-step non-parametric approach. In the first step, Wilcoxon Signed Rank tests were run separately for the two tasks and confirmed that, similarly to mean beta power, the within-block increase of beta modulation depth over the Left and Frontal ROIs was larger in the first compared to the third block for both ROT and MOT (Left ROI, ROT: Z = − 2.84, *p* = 0.01; MOT: Z = 2.55, *p* = 0.01; Frontal ROI, ROT: Z = 2.19, *p* = 0.03; MOT: Z = 2.27, *p* = 0.02) (Supplementary Table S2). These results are consistent with the findings regarding mean beta power and suggest that the within-block increase in beta oscillatory activity is maximal in the first block of both tasks with the possible presence of a ceiling effect in the last block.

In the second analysis step, we ascertained whether the observed beta modulation changes were similar in the two tasks. Independent-sample Kruskal–Wallis tests highlighted that, while in the first block the two tasks had comparable beta modulation growth in all the three ROIs, ROT3 showed greater increase than MOT3 in the Frontal ROI only (mean rank: ROT3 = 22.24, MOT3 = 14.23, H = 4.442 *p* = 0.035, Supplementary Table S2, Supplementary Figure S2).

Altogether, these results indicate that, in both ROT and MOT tasks, the within-block increases of beta power over frontal and parietal areas may follow a logarithmic trend, with a greater increase in the first block of practice and a lesser one in further training. Moreover, extensive practice of visuo-motor adaptation induces greater within-block increases of beta modulation over the frontal region, an increase that might reflect not only motor practice per se, but also learning-related processes. However, all our analyses did not show any significant correlation between adaptation rate and the practice-related beta modulation increase both in ROT1 (difference between first and last sets) (left: rho = 0.13, *p* = 0.54; frontal: rho = 0.15, *p* = 0.48; right: rho = 0.33, *p* = 0.11) and in ROT3 (left: rho = 0.21, *p* = 0.31; frontal: rho = 0.25, *p* = 0.23; right: rho = − 0.28, *p* = 0.18).

### Simple motor practice and visuo-motor adaptation differentially affect frontal increase of beta modulation in a subsequent motor test

We then compared *mov* tests, each composed by 96 movements presented at three distances and eight directions, performed at baseline (*mov*0) and after the third block (*mov*3) of both practiced tasks. *mov* tests were performed to determine whether, compared to MOT, the continuous visuomotor adaptation learning required by ROT led to performance deterioration and induced local changes in movement-related beta modulation during simple reaching movements.

Therefore, we first focused on the performance in both *mov*0 and *mov*3 (see Table 1). While no significant differences were found for peak velocity, reaction and movement times, there was a significant effect of task for directional errors. Nonetheless, inspection of the data showed a rather small effect size (η^2^ *p* = 0.097) and between tasks differences less than 1°, limiting practical relevance of this result.

**Table 1:** *mov* performance indices.

	Reaction time (ms)	Peak velocity (cm/s)	Movement time (ms)	Directional error (°)	% Correct movements
ROT*mov*0	293.4 ± 28.0	57.3 ± 11.4	256.6 ± 46.5	3.8 ± 0.6	81.8 ± 3.8
ROT*mov*3	293.6 ± 28.0	57.5 ± 11.1	257.5 ± 41.8	4.2 ± 0.7	68.1 ± 10.1
MOT*mov*0	303.4 ± 29.5	55.3 ± 11.0	281.3 ± 47.0	3.5 ± 0.5	82.4 ± 8.3
MOT*mov*3	310.7 ± 28.7	53.0 ± 10.1	283.7 ± 49.4	3.7 ± 0.7	86.3 ± 8.3
**Task df (1, 39)**					
F	2.325	0.943	3.133	4.185	19.683
* p*	0.135	0.337	0.085	**0.048**	** < 0.001**
η^2^ *p*	0.056	0.024	0.074	0.097	0.335
**Block df (1, 39)**					
F	1.803	0.740	0.154	3.578	10.615
* p*	0.187	0.395	0.697	0.066	**0.002**
η^2^ *p*	0.044	0.019	0.004	0.084	0.214
Task * Block df (1, 1)					
F	1.589	1.081	0.034	0.913	35.035
* p*	0.215	0.305	0.856	0.354	** < 0.001**
η^2^ *p*	0.039	0.027	0.001	0.023	0.473

Top: Mean ± standard deviations (SD) for each *mov* test. Bottom: Results of mixed-model ANOVAs ascertaining the effects of block and preceding task on behavioral indices. Significant differences are reported in bold (p < 0.05). F, test statistics; *p,* *p* values; η^2^ *p,* Partial Eta Squared.

The number of correct numbers*,* defined as those movements with reaction time, normalized hand path area and directional error values falling within 1.5 SD of the baseline mean *mov*0. Using a mixed-model ANOVA because of the normal distribution, we found significant main effects of Block and Task with a significant Block X Task interaction (Table 1). Indeed, while performance levels in MOT*mov* and ROT*mov* were comparable at baseline (mean ± SD, MOT*mov*0: 82.38% ± 8.33; ROT*mov*0: 81.81% ± 3.80, Table 1), after three blocks, only after ROT practice, the number of correct movements in *mov* significantly decreased (ROT*mov*3: 68.13% ± 10.09, MOT*mov*3: 86.35% ± 8.31; *see* Table 1).

These results suggest that the additional learning present in ROT but not in MOT might have induced some *neuronal overload*. In turn, in agreement with previous results^14,15^, this might have led to performance deterioration.

In the ROT task, beta modulation displayed greater increase over the frontal region compared to MOT. Thus, we next ascertained whether this was also the case during the ensuing *mov* test.

**Figure 3:**
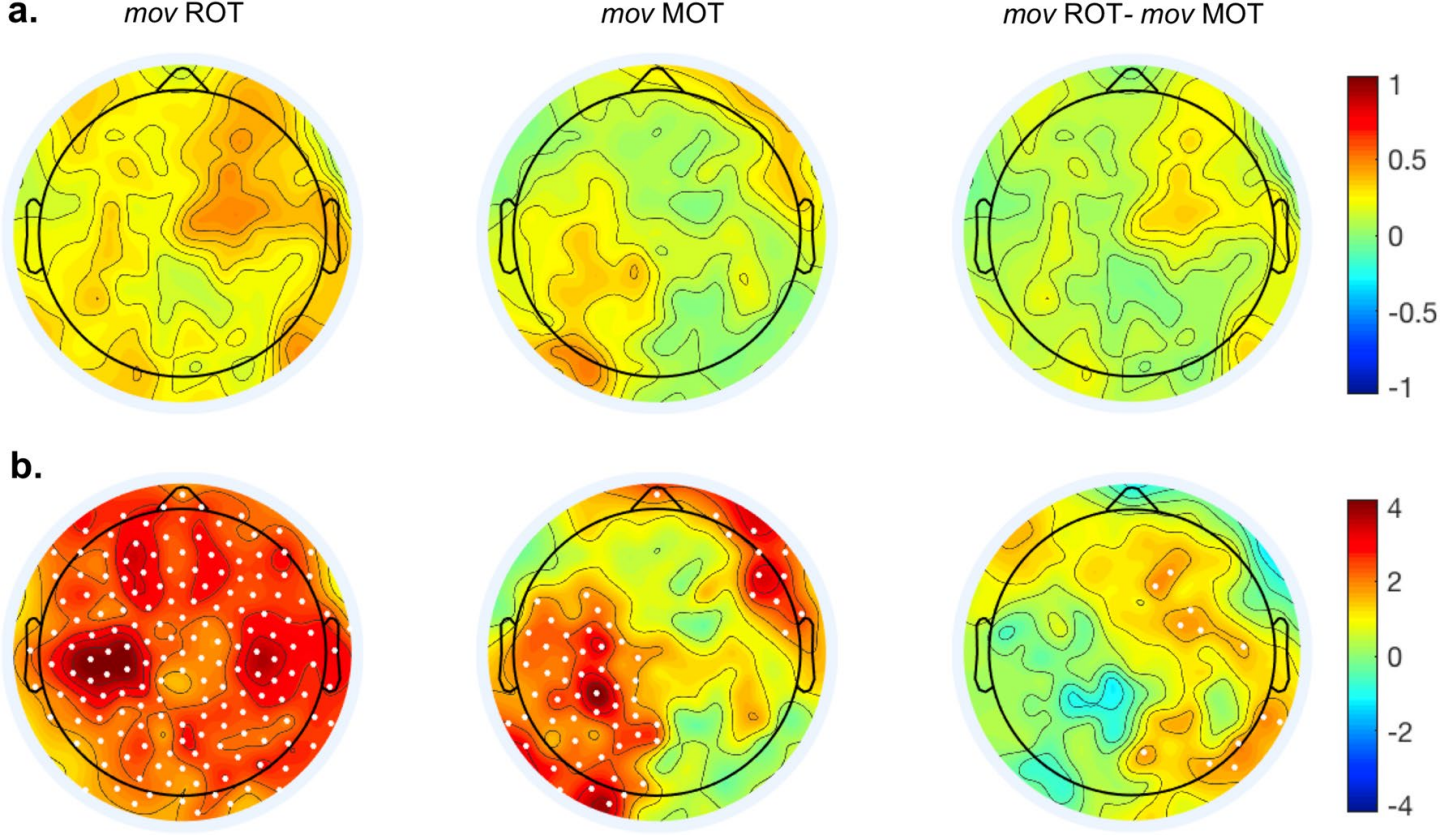
Within-block changes of average beta oscillatory activity in *mov*. (**a**) Topographic distribution of the between-block changes—computed as the difference between *mov*0 and *mov*3- in both ROT*mov* and MOT*mov*, and their differences (ROT*mov* vs. MOT*mov)*. (**b**) Maps of the Bonferroni-corrected permutation t-values comparing the between-blocks changes in ROT*mov,* MOT*mov* and their relative difference. Significant electrodes are reported as white dots (*p* ≤ 0.05).

We first determined whether the average beta oscillatory activity increased in *mov* and whether such increase differed according to the practiced task. Non-parametric permutation analyses (alpha = 0.05) on the difference between *mov*3 and *mov*0 showed a strong increase of the average beta power for both ROT*mov* (mean ± SD: 15.3% ± 21.0%, t = 2.52, *p* = 0.009) and MOT*mov* (27.1% ± 21.0%; t = 2.40, *p* = 0.02). Importantly, the direct comparison of ROT*mov* and MOT*mov* with independent-samples t-test permutation analysis showed greater power increase in ROT*mov* (mean ± SD: 36.4% ± 52.3%) compared to MOT*mov* (1.6 ± 25.0%; t = 1.74, *p* = 0.03) over frontal and right centroparietal regions (Fig. 3).

**Figure 4:**
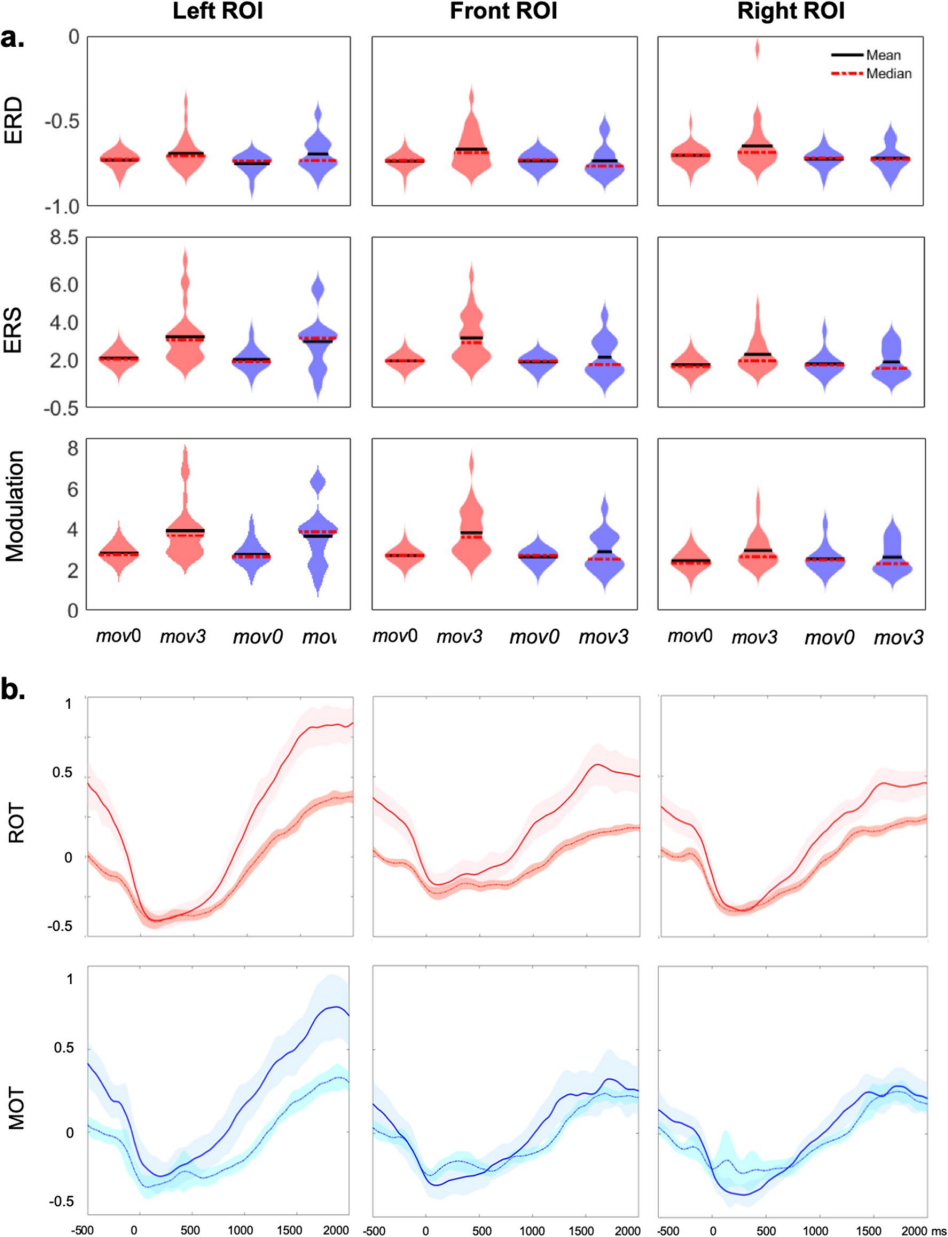
(**a**) Violin plots showing the data distribution and probability density of ROT*mov* (in red) and MOT*mov* (in violet). (**b**) Time-course of beta oscillatory activity during ROT*mov* (in red) and MOT*mov* (in blue) for Block 1 (thin, dotted line) and Block 3 (thick, solid line)**.**

We then explored the between-blocks (*mov*3 vs. *mov*0) and between-groups (ROT*mov* vs. MOT*mov*) differences in beta modulation, ERD and ERS amplitudes in the three ROIs (Left, Right and Frontal, see “Methods” section). Again, because of the normality violation, a two-step non-parametric approach was adopted. In the first step, Wilcoxon Signed Ranks Tests were run to test practice-related changes (*mov*3–*mov*0) for each ROI and session (ROT*mov* and MOT*mov*). In agreement with a previous publication^15^, in ROT*mov*, the amplitude of beta modulation and ERS significantly increased from *mov*0 to *mov*3 over the three ROIs, while beta ERD amplitude was reduced over the Right and Frontal ROIs (Fig. 4). All these changes were accompanied by an increase of mean beta power in the three ROIs (Table 2).

**Table 2:** Comparison of beta ERD, ERS, modulation depth and mean power magnitude during the baseline (*mov*0) and third block (*mov*3) in ROT*mov* and MOT*mov.*

	ERD		ERS		Modulation depth		Average power	
	ROT *mov*	MOT *mov*	ROT *mov*	MOT *mov*	ROT *mov*	MOT *mov*	ROT *mov*	MOT *mov*
**Left ROI**								
Z	1.946	1.412	3.892	2.668	3.988	2.605	3.700	2.103
*p*	0.052	0.158	**0.002**	**0.008**	**0.002**	**0.009**	**0.002**	**0.035**
$\tilde{\upmu }$ 0	− 0.725	− 0.744	2.054	1.921	2.760	2.650	0.026	0.020
$\tilde{\upmu }$ 3	− 0.705	− 0.731	3.061	3.159	3.690	3.875	0.245	0.397
**Right ROI**								
Z	2.066	0.345	3.171	0.157	3.051	0.094	2.715	0.345
*p*	**0.039**	0.730	**0.002**	0.875	**0.002**	0.925	**0.007**	0.730
$\tilde{\upmu }$ 0	− 0.699	− 0.718	1.671	1.748	2.330	2.485	0.018	0.014
$\tilde{\upmu }$ 3	− 0.684	− 0.724	1.970	1.567	2.640	2.305	0.109	− 0.005
Z	2.955	− 0.408	4.084	0.345	3.950	0.471	2.330	0.408
**Front ROI**								
*p*	**0.003**	0.683	**0.002**	0.730	**0.002**	0.638	**0.020**	0.683
$\tilde{\upmu }$ 0	− 0.731	− 0.727	1.973	1.953	2.720	2.705	0.013	0.014
$\tilde{\upmu }$ 3	− 0.686	− 0.764	2.918	1.768	3.600	2.525	0.094	0.024

Significant differences are reported in bold (p < 0.05).

Results of related-samples Wilcoxon Signed Rank Test on the differences between the two blocks (*mov*3 vs. *mov*0) in each ROI and group (ROT*mov* and MOT*mov*). Z, test statistic; *p,* *p* values; $\tilde{\upmu }$, median.

On the other hand, the same analyses on MOT*mov* revealed that the amplitude of beta modulation and ERS, as well as the beta average power, increased only over the Left ROI (Table 2, Fig. 3). These results indicate the occurrence of cumulative effect of practice in the subsequent reaching test; specifically, extended motor practice with negligible learning mostly affect beta activity over the left centro-parietal region whereas continuous visuo-motor adaptation, such as that taking place in ROT, is accompanied by an additional increase of beta modulation over the frontal and, partially, over the right region (Table 2). Accordingly, these local changes in beta modulation depth might reflect use-dependent changes in the sensorimotor, visual and frontal areas, with the first area similarly active in both tasks and the latter being more engaged during visuo-motor rotation. Of note, as per the task, correlation analyses between *mov*3 beta modulation and ROT3 adaptation rate did not yield significant results (left: rho = 0.23, *p* = 0.28; frontal: rho = − 0.08, *p* = 0.72; right: rho = − 0.24, *p* = 0.26), suggesting that that beta modulation per se does not reflect adaptation rate.

We confirmed the difference between ROT*mov* and MOT*mov* in the second step, with a direct comparison of *mov*3–*mov*0 changes between the two groups by means of independent-sample Kruskal–Wallis tests. Compared to MOT*mov*, ROT*mov* showed greater practice-related changes for both beta modulation (H = 5.46, *p* = 0.019) and ERS (H = 5.59, *p* = 0.018) in the frontal ROI, and in the right ROI for the ERS only (H = 4.02, *p* = 0.045). No differences were observed between the two groups in the average beta power (Table 3). Of note, we did not find a significant correlation between tiredness scores and beta modulation magnitudes both for ROT*mov* 3 and MOT*mov* 3 (ROT*mov*: Left ROI: rho = − 0.046 *p* = 0.82; Frontal ROI: rho = − 0.22 *p* = 0.28; Right ROI: rho = − 0.052 *p* = 0.80; MOT*mov*: Left ROI: rho = 0.01 *p* = 0.98; Frontal ROI: rho = − 0.05 *p* = 0.88; Right ROI: rho = − 0.43 *p* = 0.14).

**Table 3:** Comparison of between-blocks (*mov*3–*mov*0) ROT*mov* and MOT*mov* differences in beta ERD, ERS, modulation depth and mean power magnitude with independent-samples Kruskal–Wallis test for each ROI.

	ERD	ERS	Modulation depth	Average power
**Left ROI**				
H	0.11	0.05	0.00	0.59
*p*	0.74	0.83	1.00	0.44
μR ROT*mov*	21.44	20.70	21.00	19.96
μR MOT*mov*	20.14	21.57	21.00	23.00
**Right ROI**				
H	0.98	4.03	3.19	2.12
*p*	0.32	**0.05**	0.07	0.15
μR ROT*mov*	22.33	23.70	23.41	22.96
μR MOT*mov*	18.43	15.79	16.36	17.21
**Frontal ROI**				
H	4.72	5.59	5.46	2.04
*p*	**0.03**	**0.02**	**0.02**	0.15
μR ROT*mov*	23.93	24.19	24.15	22.93
μR MOT*mov*	15.36	14.86	14.93	17.29

Significant differences are reported in bold (p < 0.05).

Degrees of freedom = 1 in all cases. H, test statistic; *p,* *p* values; μR, rank mean.

**Figure 5:**
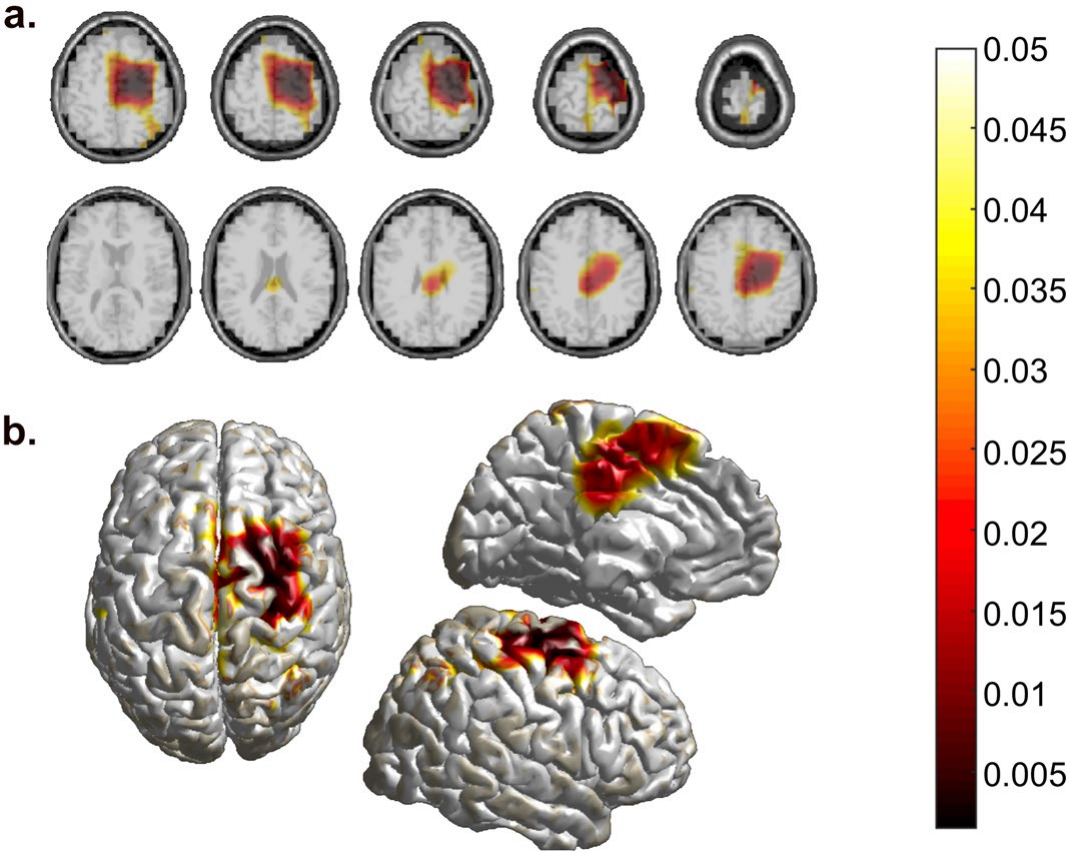
Bonferroni-corrected *p*-value distribution of Monte Carlo non-parametric permutation statistics on ROT*mov* and MOT*mov* source-reconstruction differences in between-blocks (*mov*3*–mov*0) post-movement beta activity increase. (**a**) horizontal slices were obtained by interpolating the statistics onto the MRI template. (**b**) surface plots were obtained by interpolation onto the MNI template.

Finally, we applied the *beamformer* DICS technique to estimate the sources responsible of the observed practice-related beta modulation changes between ROT*mov* and MOT*mov*. Non-parametric permutation test with Bonferroni correction was run to compare beta amplitude changes (*mov*3–*mov*0) between ROT*mov* and MOT*mov* sources. Importantly, as the channel-related results suggested that beta modulation depth changes were largely dependent from the increased ERS, source reconstruction focused on the post movement time window only (from 700 to 2000 ms, see “Methods” section). Based on the human Brainnetome atlas parcellation, ROT*mov* was associated with greater post-movement beta increase in the right precentral (i.e., primary motor cortex) and right postcentral gyrus (i.e., primary somatosensory area), right medial and dorsolateral superior frontal gyrus, right middle frontal gyrus and middle cingulate gyrus bilaterally (Fig. 5, Table 4). Interestingly, these areas are involved in the processes related in visuo-motor adaptation, as shown by previous imaging studies^16,17^.

**Table 4:** Monte Carlo non-parametric permutation statistics on the difference between ROT*mov* and MOT*mov* between-blocks (*mov*3–*mov*0) post-movement beta power increase.

Area	Brainnetome atlas label	voxels %	t (M)	t (SD)	*p* (M)	*p* (SD)
Superior Frontal Gyrus	R SFG, A8m	91.42	1.85	0.17	0.03	0.01
	L SFG, A8m	87.05	1.69	0.08	0.04	0.01
	R SFG, A6dl	98.35	2.42	0.13	0.01	0.00
	R SFG, A6m	99.87	2.27	0.17	0.01	0.01
	L SFG, A6m	70.27	1.69	0.23	0.05	0.03
Middle Frontal Gyrus	R MFG, A6vl	98.57	2.14	0.23	0.01	0.01
Precentral Gyrus	R PrG, A6cdl	93.78	2.26	0.34	0.01	0.02
	R PrG, A4ul	99.80	2.31	0.19	0.01	0.01
	R PrG, A4t	96.95	2.10	0.23	0.02	0.01
Paracentral Lobule	R PCL, A1/2/3ll	91.71	1.75	0.13	0.04	0.01
	R PCL, A4ll	99.81	2.03	0.19	0.02	0.01
	L PCL, A4ll	73.16	1.71	0.15	0.04	0.02
Postcentral Gyrus	R PoG, A1/2/3tru	77.51	1.99	0.29	0.04	0.03
Cingulate Gyrus	R CG, A24cd	72.17	1.85	0.23	0.04	0.02
	R CG, A23c	100	2.11	0.20	0.02	0.01
	L CG, A23c	85.49	1.82	0.17	0.03	0.02

Bonferroni correction was applied to address the multiple comparisons problem. Only significant sources according to the Brainnetome atlas are reported. Voxel % refers to the percentage of significant voxels for the corresponding area; M and SD are respectively the mean and standard deviation of t and *p* values.

## Discussion

The present study shows that extended motor practice, independently of the learning load, leaves traces in movement-related beta modulation of a subsequent simple reaching task, albeit with topographies and magnitudes that reflect the differential engagement of the examined ROIs in the specific tasks. Also, the finding of a greater beta modulation increase over the frontal area after the visuo-motor learning task suggests that such traces may result from the combined footprint of motor practice per se, a characteristic common to the two tasks, and of the learning-related processes, which mostly occur during visuo-motor adaptation. Altogether, these findings support the hypothesis that the traces the task left on beta modulation in the following motor test may express phenomena related to the local “usage”, as discussed in the following paragraphs.

Beta modulation increases were present in *mov*, a simple reaching test, not only after extensive practice in a visuo-motor adaptation task, ROT, as in previous work^15^, but also after practice in a motor task, MOT, without relevant learning components. However, these increases differed in terms of local magnitude: significant enhancement of beta modulation occurred over all three ROIs after ROT, but only over the Left ROI after MOT. Therefore, such increases of beta modulation could be considered as traces of two tasks’ activity in the ensuing *mov*. Indeed, these task-related differences already emerged during the ROT and MOT practice: while the degree of within-block increase over the three ROIs was similar during ROT1 and MOT1 tasks, beta modulation in the last block increased only over the frontal region during ROT3 and such an increase was greater than the corresponding one in MOT3. Importantly, these patterns of increase were mostly related to changes in beta ERS. The source reconstruction revealed that the estimated sources of the observed frontal ERS increase in ROT*mov*3 were localized in the right primary sensorimotor cortices, the pre-supplemental area and the middle region of the cingulate cortex. Indeed, the involvement of these sources to visuo- rotation needs to be confirmed with other approaches in future studies. Nevertheless, they are compatible with the findings of studies on the distribution of ERS with neuroimaging and intracranial EEG recordings^19–22^. Beta ERS recorded over the frontal region has been associated to several functions supporting motor learning, such as the maintenance of sensorimotor representations^13,23–27^, processing of sensory reafference^28,29^ and visuomotor attention^3,30^. In particular, the frontal region is highly engaged during motor adaptation to visual rotations and the superior frontal gyrus, which likely corresponds to the pre-supplementary motor area, is specifically active in the earliest phases of exposure to visuo-motor rotation^17,31,32^. Indeed, our subjects were intentionally kept in the earliest stages of adaptation, with a new visual rotation introduced every two sets of movements. Our source analysis also indicated that ROT*mov* has a greater engagement of the right parietal regions, in line with their role in visual attention tasks^33^ and previous findings implicating the right hemisphere in visuo-motor adaptation^16,17,32,34^. Therefore, it is possible that, compared to the negligible learning load in MOT, the constantly greater engagement of these regions for visuo-motor adaptation might have led to higher beta activity over the frontal and right regions. It is not clear whether or not this greater activity is directly related to learning and plasticity-related phenomena. Nevertheless, a recent fMRI study^31^ showed that while visuo-motor adaptation learning was associated with greater activity in the cerebellum, parietal and frontal areas, the activity of the right superior frontal gyrus was the only one not correlated with the magnitude of after-effects, thus suggesting that activity in this area may be not be linked to specific learning indices. In any case, the participation of right hemisphere and their specific significance in rotation learning needs to be confirmed with other approaches in future studies.

The hypothesis that beta modulation practice-related enhancements over the frontal region might reflect an increase in general processes not specifically related to learning comes from a series of consideration. While ROT and MOT share the activity of the sensorimotor network, compared to MOT, ROT adaptation involve increased activity of pre-supplementary motor area and right parietal regions^17^. Therefore, the greater enhancement of beta modulation after ROT than after MOT only in the frontal ROI (an area specifically involved in ROT learning) with equal increase in the other two areas, suggests that such enhancement of beta modulation could represent general processes involved in both usage and learning, such as energy-related ones, rather than those specifically related to LTP.

Other support to this hypothesis comes from the fact that frontal beta modulation increase after the visuo-motor adaptation practice was not paralleled by specific improvement in the task performance^14^ and that the carry-over effect in the subsequent test was accompanied by performance deterioration. We could speculate that the practice-related beta modulation and ERS increases may reflect local build-up of neuronal processes due to intensive use and normally accompanied by depletion of metabolic resources and persistent changes in ionic concentrations, as suggested by experimental evidence in humans and animals. Studies in humans have shown that, while sleep is needed to consolidate performance and to return practice-related local theta power increases to baseline levels^14^, a period of quiet rest is sufficient to restore practice-related beta power increases not only at rest^14^ but also during movement-related beta modulation^15^. In addition, studies in rodents have shown that increases of beta power in both frontal and somatosensory areas during active wake occur in parallel with local increases, in the same areas, of lactate concentration, an integrative measure of energy consumption^35^. Indeed, ex-vivo slice studies directly linked lactate-concentration to the disruption of the neuronal excitation-inhibition balance, specifically by reducing gamma-band rhythm due to transient ATP-shortage in fast-spiking inhibitory interneurons^36^. Interestingly, sharp wave-ripples, lower-energy expenditure oscillations characterizing waking immobility and slow-wave sleep, were not perturbed by lactate injection^37^. Therefore, it is possible that the local increases of beta ERS and movement-related modulation due to extensive motor practice may reflect the rise of energy consumption that is needed, in both MOT and ROT, for the buildup of neuronal activity and, in ROT, for the additional induction of early phases of LTP processes^38^. Of course, this remains for the moment only a hypothesis that needs confirmation by a series of targeted studies in humans with a multimodal approach.

In conclusion, our study confirms that extended motor practice in general leaves traces in the movement-related beta modulation of a subsequent simple motor test. Moreover, compared to simple motor practice, continuous motor adaptation to novel visual rotations is specifically associated with greater beta modulation depth increase over the frontal regions, an increase that is carried over on subsequent simple reaching movements. The observed effect might reflect local neuronal use-dependent processes likely linked to energy consumption needed for neuronal activity and for induction of LTP processes related to learning. Reduced beta modulation increase might thus represent a state of reduced energy availability and, in a second instance, a decreased capacity for plasticity^46^.

## Methods

### Participants

Two groups of subjects were recruited for this study: 28 participants (mean ± SD: 24.38 ± 3.96 years, 16 women) completed the ROT task session and 14 subjects (mean ± SD: 24.99 ± 5.43 years, 10 women) participated to the MOT task session (see below). Data from part of these subjects were presented in previous publication^14,15^.

All subjects were right-handed, had normal or corrected vision and no history of disorders affecting the nervous system. The study was approved by the CUNY University Integrated Institutional Review Board (UI-IRB) and registered with the US Department of Health and Human Services Office for Human Research Protections. The experiment was performed in accordance with the ethical principles of the Declaration of Helsinki and its subsequent amendments. Each participant signed an IRB-approved informed consent form before completing the experiment.

### Experimental design

Participants were comfortably seated in a sound-shielded room in front of a computer display and fitted with a 256-channel EEG Geodesic Sensor Net (Electrical Geodesics Inc., Eugene, OR).

Both the ROT and MOT groups underwent a baseline assessment (*mov*0) before and after (*mov*3) completing either three one-hour blocks of ROT, an implicit motor learning task, or MOT, a control task with the same features of ROT but with negligible learning components (Fig. 1a,b). At baseline and after each practice block, participants were asked to report their subjective tiredness level on a scale from 0 (i.e. no tiredness) to 10 (i.e. extreme tiredness).

### Tasks and test

The testing apparatus and the instructions to the subjects were the same for the two tasks (ROT and MOT) and the test (*mov*) and are detailed in previous publications^10,14,15^. Briefly, in all tasks and test, subjects performed out-and-back movements on a digitizing tablet starting from a central starting point to a target presented from 400 ms as a blackening circle on a screen placed in front of the subjects. Instructions were to move after the target presentation, as soon and fast as possible, without corrections and to reverse direction within the target circle without stopping. The cursor position on the screen and the central starting point were always visible. Target presentation was random in all tasks and test.

The tasks and the test differed in the following characteristics: (1) the time interval between two target presentations was 3 s in the test and 1.5 s in the two tasks; (2) in the *mov* test, the targets were presented at three distances (4, 7 and 10 cm) in eight directions (45° separation) with their radius varying with their distance from the center (0.5 cm, 0.88 cm, 1.25 cm, respectively) (Fig. 1b). A total of 96 targets were presented in each *mov* test.

In the two tasks, ROT and MOT, the target array consisted of eight, radially arranged, empty circles, all at 4 cm from a center point. In order to probe implicit learning processes, in ROT the direction of the cursor on the screen was rotated relative to the direction of the hand on the tablet in steps of 10°, 20° or 30° each, either clockwise or counterclockwise, starting from 0° (no rotation) up to a maximum of 60°. We used rather small rotation steps (maximum of 30°, either clockwise or counterclockwise) and a short time interval between successive targets’ presentation (1.5 s) to avoid awareness and implementation of cognitive strategies. Also, this protocol was designed to promote continuous learning and implicit “learning how to learn” rather than the learning of a specific visuo-motor rotation.

The imposed rotation for the ROT sets were: Block 1: 0°, 10°, 20°, 30°, 40°, 50°, 60°, 50°, 40°, 30°, 20°, 10°, 0°; Block 3: 0°, *10°, 10°, *20°, 0°, *30°, *10°, 20°, *10°, 0° (* indicates that the rotation was in the direction counter to the one applied in Block 1, which could have been either clockwise or counter-clockwise).

For each block, we ran 21 sets for ROT and 20 sets for MOT of 56 reaching movements with 30 s inter-set interval. Crucially, all three ROT blocks ended with 112 movements without rotations to avoid carry-over effects on the subsequent *mov* test. In the MOT task all the sets had no imposed rotation.

### Behavioral data recording and analyses

The (x,y) coordinates of each movement’s trajectory were recorded with a custom-designed software by E.T.T. s.r.l., MotorTaskManager, Genoa, Italy (http://www.ettsolutions.com) and analyzed using an ad-hoc Matlab-based pipeline. First, we filtered the coordinates with a Butterworth filter and then computed the first, second and third derivative of the trajectory to obtain velocity, acceleration and jerk for all the movements. Following previous publications^16,32,39^, several measures were computed for each movement; in this study we focused on: reaction time (time from target appearance to movement onset), movement time (duration of the outgoing movement), amplitude of peak velocity and directional error.

To ascertain that adaptation learning consistently occurred across ROT blocks we computed the adaptation rate based on the average set directional error (Dir Err) as: *%Adaptation* = [1 − (average DirErr/imposed rotation)] * 100. Mean adaptation rate of a block was defined as the average of all sets in that block. For both blocks (ROT1, ROT3), adaptation rates were correlated with subjective tiredness score of the corresponding blocks with Spearman rank-order correlations. Further, differences in tiredness score between the ROT and MOT Tasks and blocks (Block 1 and Block 3) were assessed with Wilcoxon signed-ranked test and Kruskal–Wallis test, respectively.

For *mov* only, we computed for each subject the percentage of correct movements based on individual performance indices during the baseline *mov*0. These indices also included the hand-path area, i.e. the area within the hand-path normalized by path length. Therefore, correct movements were defined as those with values of directional error, reaction and movement times, as well as normalized hand path area within 1.5 SD of *mov*0.

Importantly, to allow for a proper task comparison unbiased by the rotation learning occurring in the ROT task, only the movements with 0° imposed rotation were included in the analyses. Therefore, for both the ROT and MOT tasks, we extracted the kinematic characteristics of the movements included in the first and last two sets of each block (ROT1, ROT3, MOT1, MOT3).

### EEG recording and preprocessing

High density (HD) EEG data were acquired using a 256-channel HydroCel Geodesic Sensor Net (Electrical Geodesic Inc.) with a Net Amp 300 amplifier (250 Hz sampling rate, online reference electrode: Cz) and Net Station version 5.0 software. Impedances were kept below 50 kΩ throughout the recording to preserve a good signal-to-noise ratio. The entire preprocessing was carried out using the public Matlab toolbox EEGLAB version 13.6.5b^40,41^. The EEG continuous signal was FIR filtered between 1 and 80 Hz and Notch filtered at 60 Hz (59–61 Hz).

Recordings were then segmented in 4-s epochs centered on target onset, resulting in a total of 96 epochs for *mov*, and 1176 epochs for ROT and 1120 MOT tasks. A manual visual inspection of the data was carried out to remove epochs and channels containing sporadic artifacts. After trial rejection, the average number of trials per subject was 70.09 ± 18.93 and 77.75 ± 13.50 for ROT*mov* and MOT*mov*, respectively. For the ROT and MOT tasks, we kept an average of 1001.24 ± 93.28 epochs for ROT and 941.38 ± 87.52 epochs for MOT.

Independent Component Analysis (ICA) with Principal Component Analysis (PCA)-based dimension reduction (max 108 components) was applied to remove stereotypical artifacts (e.g., eye blinks, heartbeat, and muscular activity).

After a visual inspection of the power spectral density, topographical maps and time course of the estimated component for each participant, we retained an average of 16.26 ± 6.91 and 13.50 ± 3.17 components for the ROT*mov* and MOT*mov* recordings, and 16.22 ± 6.25 and 19.46 ± 7.24 components for the ROT and MOT tasks, respectively.

Electrodes rejected due to artifacts or poor signal quality were reconstructed using spherical spline interpolation, whereas those located on the cheeks and neck were removed from later analysis, resulting in 180 electrodes. Finally, the signal was re-referenced to common average.

Movements with measures outside two SD and those rejected from EEG preprocessing were excluded from further kinematic and EEG data analyses.

For the purposes of our investigation, we focused our analyses on the first and last blocks for both the test (*mov*0 and *mov*3) and the tasks (ROT/MOT1 and ROT/MOT3). All the subsequent analyses were carried out using the MATLAB Toolbox Fieldtrip^42^.

### EEG data analyses

In order to avoid confounding effects from mis-executed movements, after the preprocessing we discarded epochs representing movements whose kinematic parameters exceeded two SD and time-locked the remaining trials to movement onset (− 1 to 2.5 s).

For both tasks (ROT and MOT) and test (ROT*mov* and MOT*mov*) the signal was decomposed into their time–frequency representations by convolving the signal with complex Morlet Wavelets at linearly spaced frequencies (1–55 Hz, 0.5 Hz bins) and increasing number of cycles (3 to 10 cycles).

In the analyses of ROT and MOT tasks, we first explored whether the practice-related within-block changes in beta oscillatory activity (13.5–25 Hz) would differ between the two blocks and between the two tasks. To avoid the confounding effects of the imposed-rotation that was implemented in ROT, only the trials corresponding to zero-degree rotation were included. Thus, for both the ROT and MOT tasks, the within-block increase is represented by the difference between the last and first two zero-degree sets of each block (ROT1, MOT1, ROT3, MOT3). Importantly, for each block, the first and last trials were normalized by subtracting and dividing the average signal of the entire time-window of all trials.

As the *mov* test was implemented to assess the spectral changes occurring after extensive ROT or MOT practice, the signal was normalized by subtracting and dividing each trial by the average signal of all the trials in the entire time-window of the baseline test (*mov*0).

Because their average beta power was exceeding 2 SD of the group average we removed: three subjects from ROT, one subject from MOT and one subject from ROT*mov*. Thus, the resulting sample size was 25 and 13 subjects for the ROT and MOT tasks, and 27 and 14 subjects for ROT*mov* and MOT*mov*, respectively.

### EEG statistical analysis

#### Beta power analysis

For the ROT and MOT tasks and their respective *mov* tests (ROT*mov* and MOT*mov*), analyses on practice-related changes in average beta power were conducted with Bonferroni-corrected Monte Carlo non-parametric permutation statistics (10,000 permutations).

Non-parametric paired t-test permutation analysis on the between-blocks changes in practice-related beta power increase (Block1_last-first_ vs. Block3 _last-first_) were first run on the two tasks separately. The alpha threshold was set at 0.01 for the first block (ROT1 and MOT1) and, due to lack of statistically significant results with alpha = 0.01, at 0.05 for the third block (ROT3 and MOT3). Further, in order to unveil possible differences in the practice-related beta power increase in the two tasks, independent-samples t-test permutation analyses were run to compare Block1 (ROT1 and MOT1) and Block3 (ROT3 and MOT3) practice-related changes in the two groups (alpha = 0.05).

For the ensuing *mov* tests (ROT*mov* and MOT*mov*), the same approach was followed. Paired t-test permutation analyses (alpha = 0.05) were run to characterize between-block changes (*mov*0 vs. *mov*3) in beta oscillatory activity in the two groups separately. These analyses were followed by an unpaired t-test statistic to directly assess differences in practice-related beta power changes between the two groups (ROT*mov*0*-*ROT*mov*3 vs. MOT*mov*0*-*MOT*mov*3, alpha = 0.05).

### Beta modulation analysis

Following our previous publications^10,15^, movement-related beta modulation analyses were conducted using a personalized approach (Fig. 1).

For each participant, we run time–frequency representations within the beta frequency range (13.5–25 Hz) using Complex Morlet Wavelets at linearly spaced frequencies (0.5 Hz bins, 10 cycles) on *mov*0, normalizing the signal by the average of beta power over the entire epoch. Next, the beta ERD and ERS peak amplitude and timing were computed over three broad regions corresponding to electrodes located on the frontal, left, and right sections of the EEG net. Peak ERD was defined as the minimum value of beta power between 100 ms before movement onset to 950 ms after, whereas the peak ERS was the maximum value in the 700 to 2500 ms time range. The beta ERS-ERD peak-to-peak difference (beta modulation depth) was consequently computed for each broad region to identify the electrode with the maximum beta modulation depth and the six neighbor ones (see Supplementary Figure S3 for a topological representation of the selected ROIs for each participant). Throughout the paper, these electrode selections are denoted as Frontal, Left, and Right Regions of Interest (ROIs).

The same procedure was carried out for both ROT1 and MOT1 with the following time intervals: − 200 to 700 ms for the peak ERD and 500 to 1200 ms for the peak ERS.

For both the *mov* test and ROT/MOT tasks, time–frequency analyses were carried out on the selected ROIs (1:55 Hz, 0.5 Hz bins, 3:10 wavelet cycles) and normalized by subtracting and dividing by the average power over the entire time-window for all the trials. Peak beta ERS, ERD, modulation depth magnitude, as well as the ERS and ERD peak timing values were finally extracted for subsequent statistical analysis.

### Kinematics and mov EEG indices

In order to ascertain whether a parametric test was the appropriate statistical tool to test our data, both Shapiro–Wilks tests and Kolmogorov–Smirnov tests were run on the standardized residuals of the behavioral and EEG analyses to check for normality.

As no violation was observed for all the behavioral indices, mixed-model ANOVAs (with Blocks as within-subjects factor and Group as between-subjects factor) were run to test for any practice effect on *mov* performance indices.

For what concerns the EEG indices (peak beta ERS and ERD amplitude, beta modulation depth), as normality assumption was violated (*p* < 0.05), Wilcoxon Signed Ranks Tests were first run on ROT*mov* and MOT*mov* separately to check for blocks differences in each ROI (*mov*3 vs. *mov*0). Between-groups differences were assessed for each ROI on the difference between *mov*0 and *mov*3 (*mov*3–*mov*0) with Kruskal–Wallis Tests. Finally, Spearman’s rank correlation coefficients were computed to investigate the existence of a monotonic relationship between changes in adaptation rate, tiredness score and *mov* beta modulation magnitude.

### Source analysis

To identify the source responsible for the observed practice-related power changes, we also estimated the sources of beta oscillatory activity during a broad post-movement time window (0.7–2 s), where the beta ERS typically occurs.

For this purpose, we applied a beamforming approach, the Dynamical Imaging of Coherent Sources (DICS) method, and the estimates were calculated in the frequency domain^43^.

We first computed the cross-spectral density (CSDs) matrices of the two blocks of interest (*mov*0 and *mov*3) using multitaper spectral estimates in the beta band (13.5–25 Hz) averaged over a broad beta ERS time window (0.7–2 s).

Since individual anatomical MRIs were not collected for this study, we applied a template volume conduction model of the head based on the boundary element method (BEM), a 3-compartment (scalp, skull and brain) model provided by Fieldftrip^42^. The BEM model and standard EEG electrode positions were co-registered by projecting all electrodes to the nearest point on the head surface mesh and computing a bilinear interpolation matrix from vertices to electrodes. The bioelectric forward problem was formulated as a leadfield matrix, where each column corresponds with the potential distribution on all channels for one of the x,y,z orientation of the dipole.

Source reconstruction was performed on each subject using a spatial filter computed on the combined *mov*0 and *mov*3 CSD matrices; the resulting source was then contrasted as follows: (*mov*3–*mov*0)/*mov*0. Once each subject’s source was reconstructed, the grand-averages of ROT*mov* and MOT*mov* sources were statistically compared to highlight whether the differences observed on the channel-level could also be observed at the source level.

Non-parametric Monte Carlo permutation test with Bonferroni correction (10,000 permutations, alpha = 0.05) was applied. To identify the corresponding MNI coordinates of the significant voxels, the statistic output was interpolated with the Brainnetome Atlas^44^, a cross-validated atlas based on structural and functional connectivity measures.

## Supplementary Information

Supplementary Table S1.

Supplementary Table S2.

Supplementary Figures.
