# Author Correction: Frontal increase of beta modulation during the practice of a motor task is enhanced by visuomotor learning

**DOI:** 10.1038/s41598-021-03659-0

**Published:** 2021-12-09

**Authors:** E. Tatti, F. Ferraioli, J. Peter, T. Alalade, A. B. Nelson, S. Ricci, A. Quartarone, M. F. Ghilardi

**Affiliations:** 1 CUNY School of Medicine, 160 Convent Avenue, Harris Hall Room 008, New York, NY 10031 USA; 2 DIBRIS University of Genova, 16145 Genoa, Italy; 3 Department of Biomedical, Dental Sciences and Morphological and Functional Images, University of Messina, 98125 Messina, Italy

Correction to: *Scientific Reports* 10.1038/s41598-021-97004-0, published online 31 August 2021

The Acknowledgments section in the original version of this Article was incomplete.

“This work was supported by NIH P01 NS083514 (MFG). Kinematic data were collected with custom-designed software, MotorTaskManager, produced by E.T.T. s.r.l. We thank Martina Bossini Baroggi, Giulia Aurora Albanese and Giorgia Marchesi for the implementation of the kinematic analysis program (Marky) that was used to mark the kinematic data and Ramtin Mehraram for help in collecting and analyzing some of the MOT task data.”

now reads:

“This work was supported by NIH P01 NS083514 (MFG) and DOD W81XWH-19-1-0810 (MFG, AQ). Kinematic data were collected with custom-designed software, MotorTaskManager, produced by E.T.T. s.r.l. We thank Martina Bossini Baroggi, Giulia Aurora Albanese and Giorgia Marchesi for the implementation of the kinematic analysis program (Marky) that was used to mark the kinematic data and Ramtin Mehraram for help in collecting and analyzing some of the MOT task data.”

The original Article has been corrected.

