# Supplementary material for: Frontal increase of beta modulation during the practice of a motor task is enhanced by visuomotor learning
Source: Sci Rep. 2021 Aug 31;11:17441. doi: 10.1038/s41598-021-97004-0 (PMC8408223; doi:10.1038/s41598-021-97004-0)
Supplement: Supplementary file 1 — Supplementary Table S1. [file 41598_2021_97004_MOESM1_ESM.docx]

**Supplemental Table S1.**

| *Kruskal-Wallis Test* | | | | |  |  |  |  |  |
| --- | --- | --- | --- | --- | --- | --- | --- | --- | --- |
|  | Reaction Time | | Movement Time | | | Peak Velocity | | Directional Error | |
|  | Block 1 | Block 3 | Block 1 | Block 3 | | Block 1 | Block 3 | Block 1 | Block 3 |
| H | 0.665 | 0.148 | 1.403 | 0.227 | | 1.331 | 0.360 | 1.331 | 5.541 |
| p value | 0.415 | 0.701 | 0.236 | 0.633 | | 0.249 | 0.549 | 0.249 | **0.019** |
| μR ROT | 20.56 | 20.00 | 17.96 | 20.12 | | 21.00 | 20.28 | 21.00 | 22.56 |
| μR MOT | 17.46 | 18.54 | 22.46 | 18.31 | | 16.62 | 18.00 | 16.62 | 13.62 |

| *Wilcoxon Test* | | | |  |  |  |  |  |  |  |  |  |
| --- | --- | --- | --- | --- | --- | --- | --- | --- | --- | --- | --- | --- |
|  | Reaction Time | | | | Movement Time | | Peak Velocity | | Directional Error | |  |  |
|  | ROT | | MOT | | ROT | MOT | ROT | MOT | ROT | MOT |  |  |
| Z | -0.605 | | -0.314 | | 0.713 | -0.804 | -1.332 | -0.804 | 1.574 | -0.175 |  |  |
| p value | 0.545 | | 0.753 | | 0.476 | 0.422 | 0.183 | 0.422 | 0.115 | 0.861 |  |  |
| μ̃ 1 | 7.36 | | 5.46 | | -6.45 | 2.42 | 1.92 | 0.36 | 0.03 | -0.15 |  |  |
| μ̃ 3 | 4.20 | | 4.94 | | -0.400 | 0.31 | -0.97 | -2.08 | 0.81 | 0.11 |  |  |
|  |  |  |  | |  |  |  | | | | |  |

**Supplemental Table S1.** Practiced-related performance change between blocks (Block1 and Block 3) and tasks (ROT and MOT). All statistical comparisons were done on the difference between the last and first sets of each block (sets with 0° rotation) to characterize the effect of adaptation learning (ROT) and its control (MOT) on behavioral performance.

Top. Independent-samples Kruskal-Wallis Test (all comparisons: degrees of freedom = 1) comparing the change of performance between the two tasks, ROT and MOT in the first (Block 1) and last (Block 3) blocks. Bottom. Related-samples Wilcoxon Signed-Rank Test comparing performance of the first and last block in ROT and MOT. Rank mean and median differences of Reaction Time and Movement Time are expressed in ms and those of Peak Velocities in cm/s. (μR: rank mean; μ̃: median).
