# Supplementary material for: Frontal increase of beta modulation during the practice of a motor task is enhanced by visuomotor learning
Source: Sci Rep. 2021 Aug 31;11:17441. doi: 10.1038/s41598-021-97004-0 (PMC8408223; doi:10.1038/s41598-021-97004-0)
Supplement: Supplementary file 2 — Supplementary Table S2. [file 41598_2021_97004_MOESM2_ESM.docx]

**Supplemental Table S2**

*Wilcoxon Test*

|  | |  | ERD | | ERS | | Modulation depth | | Average power | |
| --- | --- | --- | --- | --- | --- | --- | --- | --- | --- | --- |
|  | | | ROT | MOT | ROT | MOT | ROT | MOT | ROT | MOT |
| Left ROI | Z | | -3.03 | -1.64 | -2.97 | -2.48 | -2.84 | -2.55 | -2.68 | -2.13 |
|  | p | | **0.002** | 0.10 | **0.003** | **0.01** | **0.01** | **0.01** | **0.01** | **0.03** |
|  | μ̃ 1 | | 0.01 | 0.02 | 0.44 | 0.43 | 0.41 | 0.39 | 0.17 | 0.16 |
|  | μ̃ 3 | | -0.01 | 0.01 | 0.19 | -0.02 | 0.23 | -0.03 | 0.04 | 0.03 |
| Right ROI | Z | | -2.84 | -0.45 | -2.03 | -0.38 | -1.95 | -0.38 | -3.03 | -0.38 |
|  | p | | **0.01** | 0.65 | **0.04** | 0.70 | 0.05 | 0.70 | **0.00** | 0.70 |
|  | μ̃ 1 | | 0.02 | 0.02 | 0.37 | 0.13 | 0.34 | 0.08 | 0.09 | 0.08 |
|  | μ̃ 3 | | -0.02 | 0.01 | 0.09 | 0.01 | 0.09 | 0.06 | -0.03 | 0.02 |
| Frontal ROI | Z | | -2.65 | -0.87 | -2.30 | -2.06 | -2.19 | -2.27 | -1.79 | -1.85 |
|  | P | | **0.01** | 0.38 | **0.02** | **0.04** | **0.03** | **0.02** | 0.07 | 0.06 |
|  | μ̃ 1 | | 0.04 | 0.02 | 0.56 | 0.50 | 0.50 | 0.50 | 0.18 | 0.19 |
|  | μ̃ 3 | | 0.01 | 0.01 | 0.31 | -0.02 | 0.29 | 0.00 | 0.09 | 0.07 |

*Kruskal-Wallis Test*

|  |  | | ERD | | ERS | | Modulation depth | | Average power | |
| --- | --- | --- | --- | --- | --- | --- | --- | --- | --- | --- |
|  | | | Bl 1 | Bl 3 | Bl 1 | Bl 3 | Bl 1 | Bl 3 | Bl 1 | Bl 3 |
| Left  ROI | | H | 0.20 | 4.06 | 0.00 | 2.14 | 0.04 | 3.02 | 0.13 | 0.13 |
|  |  | p | 0.65 | **0.044** | 0.99 | 0.14 | 0.84 | 0.08 | 0.72 | 0.72 |
|  |  | μR ROT | 18.92 | 16.88 | 19.48 | 21.40 | 19.24 | 21.76 | 19.04 | 19.96 |
|  |  | μR MOT | 20.62 | 24.54 | 19.54 | 15.85 | 20.00 | 15.15 | 20.39 | 18.62 |
| Right  ROI | | H | 0.01 | 4.57 | 0.32 | 0.00 | 0.40 | 0.04 | 0.00 | 3.24 |
|  |  | p | 0.94 | **0.032** | 0.57 | 0.99 | 0.53 | 0.84 | 0.99 | 0.07 |
|  |  | μR ROT | 19.40 | 16.72 | 20.24 | 19.48 | 20.32 | 19.76 | 19.48 | 17.16 |
|  |  | μR MOT | 19.69 | 24.85 | 18.08 | 19.54 | 17.92 | 19.00 | 19.54 | 24.00 |
| Frontal  ROI | | H | 1.71 | 0.09 | 0.10 | 3.70 | 0.10 | 4.44 | 0.02 | 0.17 |
|  |  | p | 0.19 | 0.77 | 0.75 | 0.05 | 0.75 | **0.035** | 0.89 | 0.68 |
|  |  | μR ROT | 21.20 | 19.88 | 19.92 | 22.00 | 19.92 | 22.24 | 19.32 | 20.04 |
|  |  | μR MOT | 16.23 | 18.77 | 18.69 | 14.69 | 18.69 | 14.23 | 19.85 | 18.46 |

**Supplemental Table S2.** Effect of practice and task (ROT and MOT) on beta ERD, ERS, modulation depth and mean power. Top. For each ROI (Left, Right, Frontal) and preceding task (ROT or MOT), Wilcoxon Signed-Rank Test assessed the effect of practice (within-blocks last-first sets) between the two blocks (Block1 and Block3). Bottom. Kruskal-Wallis Test compared practice-related changes in beta oscillatory activity indices between ROT and MOT tasks (degrees of freedom = 1 in all cases). Significant values are in bold. (μR: rank mean; μ̃: median).
