# Supplementary material for: Frontal increase of beta modulation during the practice of a motor task is enhanced by visuomotor learning
Source: Sci Rep. 2021 Aug 31;11:17441. doi: 10.1038/s41598-021-97004-0 (PMC8408223; doi:10.1038/s41598-021-97004-0)
Supplement: Supplementary file 3 — Supplementary Figures. [file 41598_2021_97004_MOESM3_ESM.pdf]

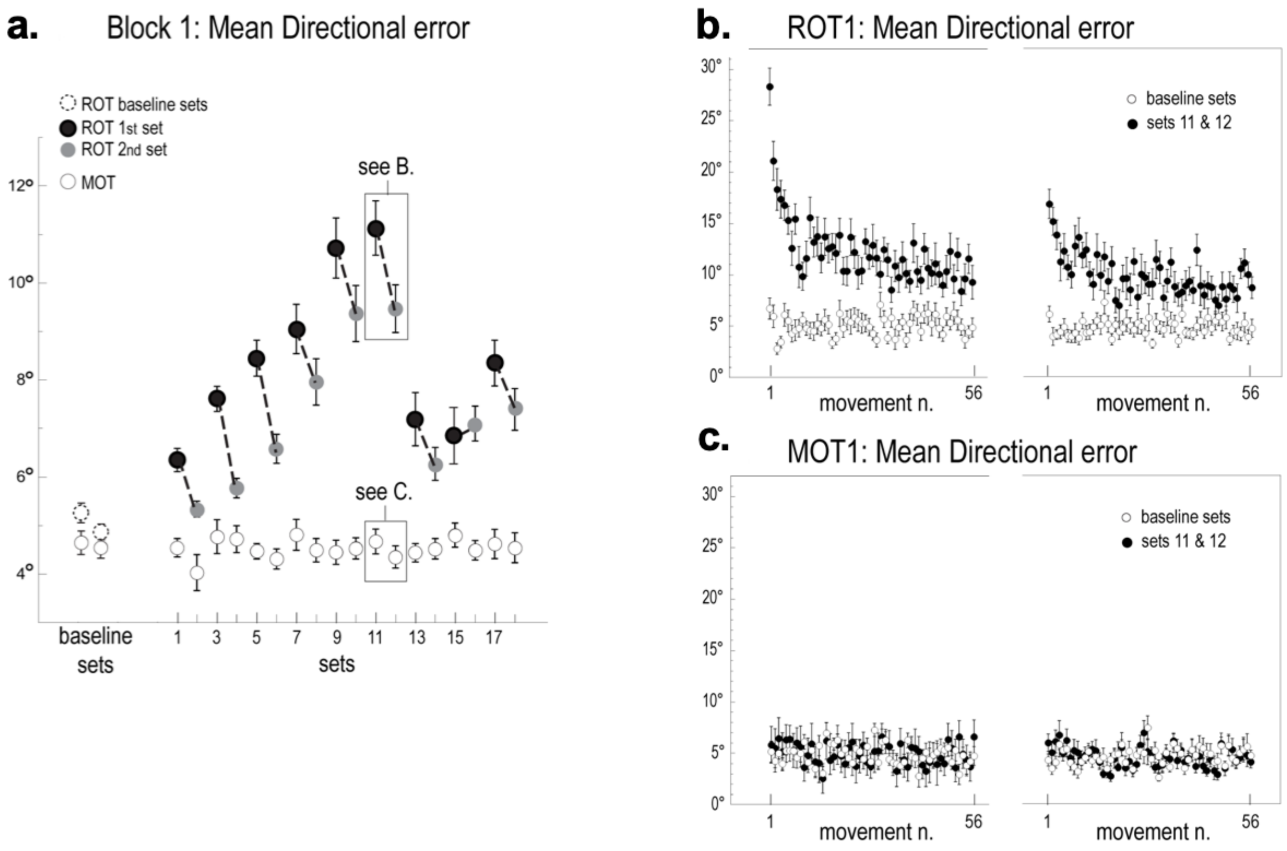

Supplementary Figure S1

a. Mean directional error of ROT1 and MOT1. Average of the directional errors for each set of ROT1 (two baseline without imposed rotation, and for each of the 10° rotation step up to 60°, see methods) and MOT1 (all sets without imposed rotation). In ROT1, mean directional error changes with different rotation steps, in agreement with the results of previous publications (Ghilardi et al., 2000; Huber et al., 2004; Krakauer et al., 2004; Perfetti et al., 2011; Moisello et al., 2015). A dotted line links the ROT1 sets with the same degree of rotation: the first of the two sets is represented with filled black dots while the second is in grey. In general, directional error decreases in the second set compared to the first one. The circles in the rectangle correspond to sets 11 and 12, where a 60° rotation was imposed (see **b.**). The average directional error in MOT1, represented with empty circles, is similar across all sets. The rectangle highlights the mean directional error of sets 11 and 12 (see **c.**).

b. Average directional errors across subjects per each of the ROT1 movement. The black dots represent the average directional errors of the two consecutive sets (sets 11 and 12 highlighted in **a.**; 56 movements each) where the rotation step of 60° was imposed (see methods). The mean directional error decreases across movements mostly in set 11, suggesting that learning has occurred. Empty dots represent the average directional errors across subjects per each movement of the two consecutive baseline sets without imposed rotation in ROT1. Differently from sets 11 and 12, the values of the directional error are rather stable across both baseline sets.

c. Average directional errors across subjects per each of the MOT1 movement of the baseline sets (empty dots, 56 movements each) as well as of the sets 11 and 12 highlighted in **a.** (black dots, 56 movements each). The Y-axis scale is the same as in **b.** for comparison with ROT1. The values of the mean directional error are rather stable across all sets.

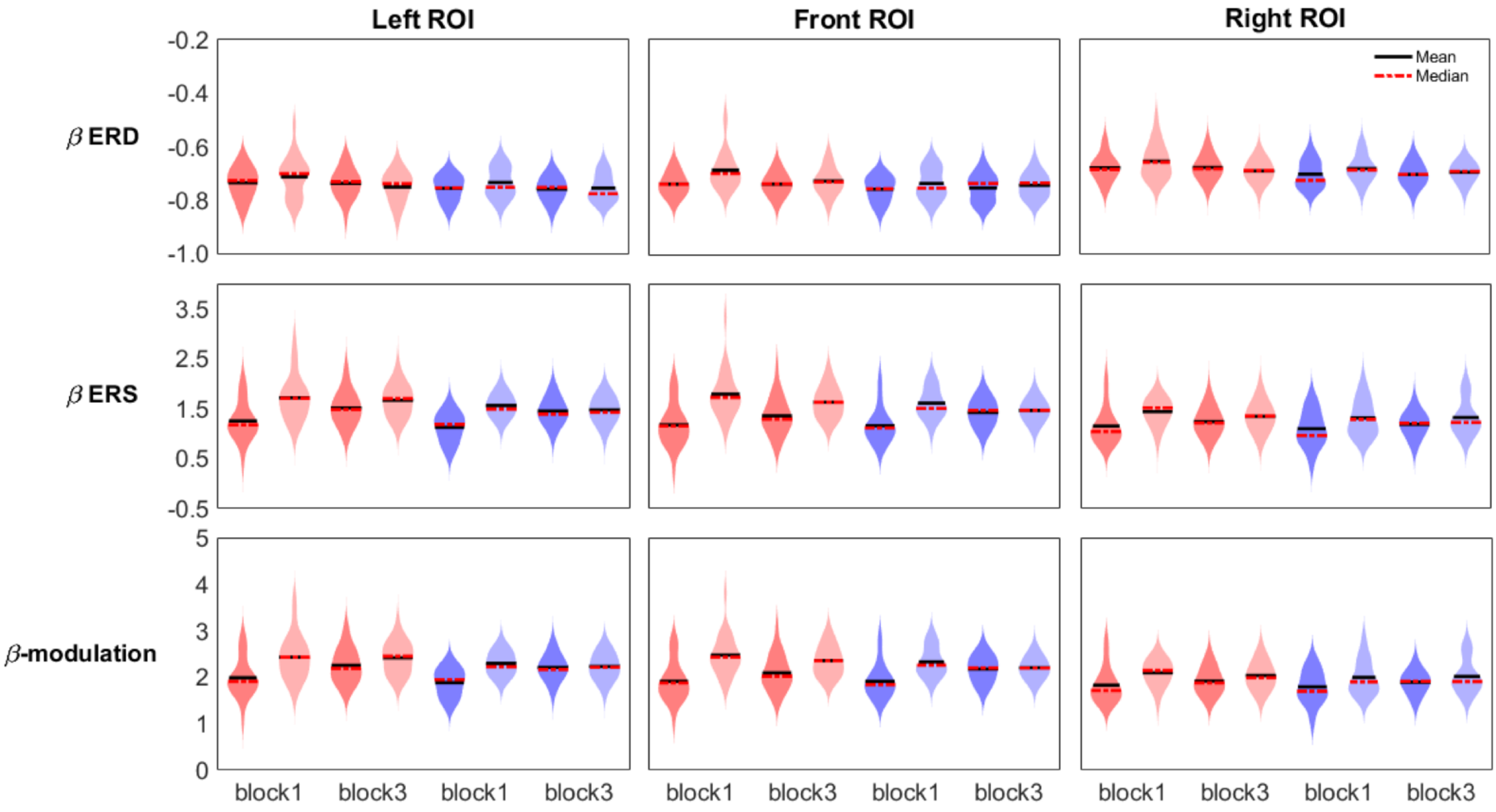

Supplementary Figure S2. Violin plots showing the data distribution and its probability density of the first (darker) and last two sets (lighter) of ROT (in red) and MOT (in violet).

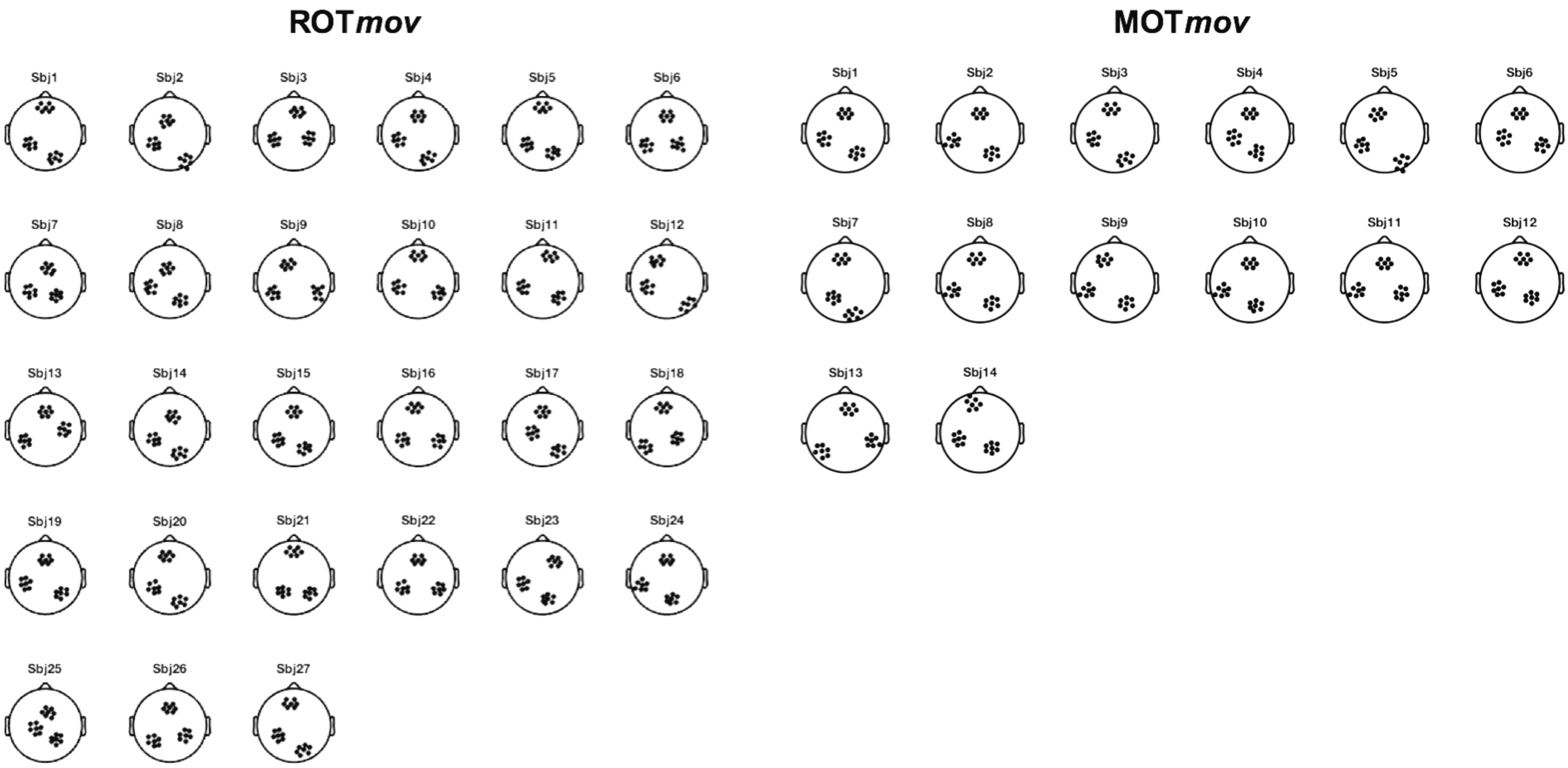

Supplementary Figure S3. Topographies showing the selected electrodes for the Left, Frontal, and Right ROIs for each subject in the ROT*mov* and MOT*mov*.
